# Psychological safety in sport: validation of the French Sport Psychological Safety Inventory (SPSI-FR)

**DOI:** 10.3389/fpsyg.2026.1855024

**Published:** 2026-06-22

**Authors:** Véronique Boudreault, Juliette Maurin, Lara Pomerleau-Fontaine, Charles-Anthony Dubeau, Francelyne Jean-Baptiste, Sophie Brassard, Jacinthe Dion, Sylvie Parent

**Affiliations:** 1 Aléo Research Chair, Université de Sherbrooke, Sherbrooke, QC, Canada; 2 Faculty of Human Kinetics, Université de Sherbrooke, Sherbrooke, QC, Canada; 3 Department of Psychology, Faculty of Humanities, Université de Sherbrooke, Sherbrooke, QC, Canada; 4 School of Psychology, Faculty of Social Sciences, Université Laval, Quebec City, QC, Canada; 5 Interdisciplinary Research Center on Intimate Relationship Problem and Sexual Abuse (CRIPCAS), Montreal, QC, Canada; 6 Department of Psychology, Université du Québec à Trois-Rivières, Trois-Rivières, QC, Canada; 7 Sexual Violence and Health Research Team (ÉVISSA), Université du Québec à Montréal, Montreal, QC, Canada; 8 Canada Research Chair in Sexual Violence Among Vulnerable Youth, Université du Québec à Trois-Rivières, Trois-Rivières, QC, Canada; 9 Department of Physical Education, Faculty of Education, Université Laval, Quebec City, QC, Canada; 10 Research Chair in Security and Integrity in Sport, Université Laval, Quebec City, QC, Canada

**Keywords:** adolescents, athletes, coach-athlete relationship, measurement, mental health, violence

## Abstract

**Introduction:**

Psychological safety is increasingly recognized as a key determinant of athletes’ wellbeing and sustainable performance, particularly in youth sport. Despite growing interest in the construct, validated tools to measure it remain largely limited to English contexts. This study thus aimed to validate a French translation of the Sport Psychological Safety Inventory (SPSI).

**Materials and methods:**

A sample of 210 francophone elite youth athletes aged between 14 and 25 (*M*_age_ = 18.2, *SD* = 2.26; 68.6% female) from a wide range of sports completed an online survey in 2024.

**Results:**

A confirmatory factor analysis, replicating the original three-factor structure of the SPSI (the factors being “mentally healthy environment”, “mental health literacy” and “low self-stigma”) showed good model fit (RMSEA = 0.076, SRMR = 0.034, CFI = 0.990, and TLI = 0.986) and strong internal consistency (*α* = 0.87; *ω* = 0.93). Furthermore, demonstrating good convergent and divergent validity, the scores of the French SPSI correlated positively with psychological wellbeing (*r* = 0.41) and quality of the coach–athlete relationship (*r* = 0.39), and negatively with interpersonal violence in sport (*r* = −0.22). Gender invariance was demonstrated, as neither scalar measurement nor latent mean scores differed between boys and girls.

**Discussion:**

These findings support the SPSI-FR as a reliable and valid measure of psychological safety in francophone sport contexts for both girls and boys. Its robust psychometric properties enable its use to monitor athlete wellbeing, assess intervention outcomes, and examine associations between psychological safety, mental health, and other sport environment variables.

## Introduction

Psychological safety is increasingly recognized as a cornerstone of athlete’s wellbeing, interpersonal functioning, and sustainable performance in sport (Vella et al., 2024). Originally defined in organizational psychology as “a shared belief that the team is safe for interpersonal risk taking” (Edmondson, 1999, p. 350), the construct has since been applied to high-performance sport. In this field, it refers to “the perception that one is protected from, or unlikely to be at risk of, psychological harm in sport” (Vella et al., 2024, p. 530). Psychologically safe environments enable athletes to raise concerns, admit mistakes, and seek help without fear of reputational damage, selection consequences, or relational backlash (Walton et al., 2024). Such environments are characterized by openness, respect, inclusivity, and proactive support systems, such as anti-harassment policies and mental health literacy initiatives that are visibly endorsed by leaders, coaches, and athletes (Walton et al., 2024).

Despite its relevance, psychological safety remains understudied in sport contexts. Walton et al. (2024) proposed one of the few sport-specific models, contrasting unsafe environments, marked by stigma, silence, and barriers to support, with safe ones, which actively foster help-seeking, accountability, and care. An important distinctive element in Walton et al.’s model is the coach-athlete relationship, which has long been identified as a key determinant of athlete’s wellbeing. When marked by trust, respect, and open communication, this relationship notably supports the athlete’s motivation, belonging, and safety (Davis et al., 2019; Jowett et al., 2023), while an authoritarian coach can heighten vulnerability to harm, silence voices, and instill a culture of fear (Stirling and Kerr, 2009).

While psychological safety is relevant across all sport levels, it may be particularly important for elite youth athletes. Indeed, elite youth athletes, typically aged between 12 and 17 years old, train in high performance programs that support their development towards Olympics, Paralympic, and professional levels (Gwyther et al., 2024). They are thus exposed to both high-performance demands and developmental factors that can influence mental health (Bergeron et al., 2015). Intensive, year-round training starting at a very young age is common in elite youth athletes from several sports, and these conditions have been associated with increased risks of overuse injuries, burnout, and psychological stress (Bergeron et al., 2024). In addition, selection processes for elite sport pathways often occur during adolescence, requiring athletes to make precipitated career-defining decisions (Bergeron et al., 2024). Furthermore, elite youth athletes must balance their high training load with their academic commitments, a combination associated with role conflict, fatigue, and elevated stress (O’Neil et al., 2021). Moreover, power asymmetries are particularly pronounced in sport, as athletes depend on coaches and sport officials for selection and access to opportunities (Walton et al., 2024). Evidence indicates that such dependencies can reduce the likelihood of reporting misconduct or seeking help, especially in environments lacking psychological safety (Mountjoy et al., 2016; Walton et al., 2024). These sports-related risk factors occur in adolescence, a period already marked by increased vulnerability to mental health difficulties. Epidemiological studies have indeed shownthat about three quarters of lifetime mental disorders emerge before age 25 (Kessler et al., 2007). Neurodevelopmental immaturity of the prefrontal cortex, combined with heightened emotional reactivity and sensitivity to social evaluation, contributes to limited self-regulation and greater susceptibility to stress (Somerville, 2013; Steinberg, 2005). These developmental factors may very well magnify the impact of interpersonal stressors and environmental pressures of elite youth athletes, making psychological safety a potentially crucial protective factor for them. Without it, athletes may internalize distress, normalize harmful behaviors, or delay help-seeking, with long-term consequences for both health and performance (Walton et al., 2024).

This intersection of developmental vulnerability and sport-specific pressures also helps explain why youth athletes may be particularly at risk of interpersonal violence in its various forms (Pankowiak et al., 2023). Interpersonal violence includes physical, psychological, sexual violence and neglect and is defined as “Interpersonal violence in sport includes physical, psychological, and sexual violence, as well as neglect; it may occur online or offline and may lead to injury, death, psychological harm, maldevelopment, or deprivation (Tuakli-Wosornu et al., 2024, p. 5). Empirical work consistently shows that violence in sport can arise from multiple sources, coaches, support staff, peers, and institutional practices, and that each operates through distinct mechanisms of harm (Tuakli-Wosornu et al., 2024). Violence between athletes is a central component of this landscape. It encompasses behaviors such as hitting, pushing, denigrating, ridiculing, coercing, or engaging in non-consensual sexual acts (Parent and Fortier, 2017). These behaviors circulate within team dynamics and can occur in contexts such as initiations, training settings, social media, team social events, locker rooms and showers, and even during games or competitions (Tuakli-Wosornu et al., 2024). Athlete-to-athlete violence is often embedded in peer hierarchies, status negotiation, and group norms, which can normalize hostile or degrading interactions and reduce the likelihood that athletes identify or report them as abusive (Tuakli-Wosornu et al., 2022). Violence perpetrated by individuals in positions of authority operates through different structural conditions. Athletes depend on these figures for selection, training, evaluation, and competitive access, which increases coercive potential, undermines disclosure, and facilitates the normalization of harmful conduct under narratives of discipline or performance optimization (Tuakli-Wosornu et al., 2024). Manifestations include psychological manipulation, punitive practices, excessive training loads, sexual exploitation, and neglect of physical or emotional wellbeing. Across both peer and authority relationships, acts of violence may appear isolated but are more often embedded within performance-driven systems shaped by “performance-at-all-costs” norms (Daignault et al., 2023; Parent and Fortier, 2017, 2018). Youth athletes are particularly vulnerable because they face strong pressures to conform to team norms to secure belonging and acceptance (Fortier et al., 2020; Fournier et al., 2022). Evidence links these environments to depression, anxiety, emotional exhaustion, disordered eating, and increased risk of sport dropout (Mountjoy et al., 2016; Tuakli-Wosornu et al., 2024). Repeated exposure to interpersonal violence, whether from authority figures or from peers, distorts athletes’ perceptions of acceptable conduct and erodes their sense of dignity and agency (Parent and Fortier, 2018).

Against this backdrop, psychological safety offers a conceptual and practical counterpoint. Environments where athletes feel secure to speak up, report misconduct, and seek support can function both as protective factors and as drivers of cultural change. This is particularly critical for elite youth athletes, whose dependence on authority figures and need for acceptance within their sport communities make them less likely to challenge harmful behaviors (Fournier et al., 2022; Hughes and Coakley, 1991). When systemic norms and leadership practices actively empower youth athletes’ voices, misconduct is more likely to be addressed, wellbeing is protected, and collective trust is reinforced (Walton et al., 2024). Ultimately, psychological safety highlights both the absence of harm and the presence of protective mechanisms. It provides a framework for understanding how sport environments can buffer against the detrimental effects of violence while also enabling athletes, particularly youth athletes in formative stages of development, to thrive and achieve sustainable performance (Vella et al., 2024). By situating athlete protection and wellbeing as inseparable from performance sustainability, psychological safety reframes the goals of sport systems toward cultures that support both excellence and humanity.

Advancing this framework requires valid and culturally appropriate instruments. A reliable measurement of psychological safety is essential to identify at-risk environments, evaluate interventions, and inform policies safeguarding athletes’ mental health and wellbeing, while enabling cross-study comparability and cumulative knowledge. To address this need, Rice et al. (2022) developed the Sport Psychological Safety Inventory (SPSI), an 11-item self-reported instrument designed to capture athletes’ perceptions of safety in their current sport environment. The SPSI was originally developed and validated with 337 elite Australian athletes from a broad range of sports (*M*_age_ ≈ 24 years) and 238 high-performance coaches and practitioners. The SPSI is grounded in the premise that psychological safety in sport is multidimensional, encompassing relational, structural, and cultural elements that shape athletes’ experiences. It thus comprises three subscales: (1) mentally healthy environment, which assesses perceptions of emotional support, openness, and non-judgment from the environment, (2) mental health literacy, which measures the visibility, accessibility, and perceived usefulness of mental health resources, and (3) low self-stigma, which captures the absence of shame or discomfort in acknowledging and addressing psychological issues. Initial validation studies in English-speaking adult athletes demonstrated the strong internal consistency of each dimension and the total SPSI score, with Cronbach’s alpha (*α*) between 0.72 and 0.92 and McDonald’s omegas (*ω*) between 0.75 and 0.92. The (original) English SPSI also showed a stable three-factor structure (RMSEA = 0.070, 90% CI [0.056, 0.085]; SRMR = 0.077; CFI = 0.935; TLI = 0.913), and meaningful associations with related constructs, with all dimensions and the total SPSI score correlating positively with psychological wellbeing and help-seeking behaviors, and negatively with self-stigma and indicators of psychological distress (Rice et al., 2022).

Beyond the original validation, subsequent studies have examined the SPSI in other cultural contexts. A Persian adaptation demonstrated acceptable psychometric properties in a sample of 220 Iranian competitive athletes (112 men, 108 women) drawn from 11 team and individual sports and spanning recreational to international levels. Confirmatory factor analysis replicated the original three-factor structure, and all subscales showed adequate reliability (coefficients > 0.70). Concurrent validity was supported through strong correlations between SPSI subscales and the total score (*r* = 0.83–0.87), as well as theoretically coherent negative associations with worry (*r* = −0.78) and competitive rumination (*r* = −0.69), reinforcing the generalizability of the SPSI’s three-factor structure beyond English-speaking settings (Molaviniya et al., 2024). More recently, a Swedish investigation with 371 elite athletes (Lundqvist et al., 2025) provided further evidence of reliability for the subscales (*ω* = 0.81–0.88) and reaffirmed the multidimensional structure, while also identifying limits to scalar invariance across gender. Although additional validation work remains necessary to ensure global applicability, these findings seem to indicate that the SPSI is cross-culturally applicable.

To date, no validated French version of the SPSI exists. This restricts its use in francophone sport settings and constrains the development of culturally grounded research. This gap is particularly salient given that sport systems may differ cross-culturally (Steele et al., 2020) in language, values, communication norms, and organizational structures, all of which can influence how psychological safety is expressed and perceived. It is also noteworthy that empirical studies on psychological safety in sport overall remain scarce, and are almost non-existent with youth elite athletes, even though this population is simultaneously exposed to developmental vulnerabilities and high-performance pressures. While the availability of the SPSI in English represents an important step for advancing this field, its lack of linguistic and cultural validation in French risks excluding francophone athletes from this emerging area of research and limiting opportunities for cross-cultural comparisons. Developing and validating a French version of the SPSI (SPSI-FR) would therefore enable precise assessment of psychological safety among francophone athletes, extend the reach of studies to previously understudied populations, and provide a robust tool to evaluate and monitor the impact of interventions aimed at improving the psychosocial climate of youth sport and ultimately to inform policies designed to safeguard the mental health and wellbeing of francophone elite youth athletes.

### Objectives of the present study

This study addressed the lack of validated tools to assess psychological safety among French-speaking elite youth athletes. In the present study, this refers specifically to Canadian elite youth athletes who completed the French-language version of the questionnaire. The study evaluated the psychometric properties of the French version of the Sport Psychological Safety Inventory (SPSI-FR), including factorial structure, internal consistency, convergent and divergent validity, and measurement invariance across gender. It also examined associations between psychological safety and theoretically related constructs, including psychological wellbeing, the coach–athlete relationship, and self-reported exposure to interpersonal violence.

## Materials and methods

### Procedure

This study is part of a larger longitudinal project examining Canadian elite youth athletes’ mental health and its moderators. The data herein presented were collected from July 2024 to November 2024, as part of the second wave of data collection of said project. Ethical approval for that project was obtained from Université de Sherbrooke’s Research Ethics Committee of Education and Social Sciences (approbation #2021-2809).

Participants of the present study were recruited from one of three strategies. First, participants from the first wave of the larger longitudinal project who had agreed to take part in the second wave were emailed by the research team to do so. Second, the authors asked provincial sport federations (PSOs) to forward an email to all eligible athletes to participate in the present study. To reduce any perceived pressure from the federations, it was clearly indicated in the email that it came from a third-party organization, the Aléo Research Chair (which oversees the larger longitudinal research). Finally, the study was also advertised on social media. Participants who completed the entire questionnaire were eligible to win one of two pairs of wireless headphones. The questionnaires were filled out on LimeSurvey and took approximately 30 min to complete.

### Participants

To be eligible in the current study, athletes needed to be (1) aged between 14 and 25 years old, and (2) identified “*élite*” or “*relève*” by their PSOs. Athletes with such label have a high potential in their sport and generally compete at a national level or above. Ice hockey players from the Quebec Maritimes Junior Hockey League were also invited to participate as they comply with the age criteria and do compete at a national level.

Of the estimated 1,800 elite youth athletes reached, 483 (approximately 25%) accessed the questionnaire. Of this number, 273 were excluded from the final sample: 34 did not give their consent; 33 completed only partially the sociodemographic data; 144 were outside the age range; 11 did not provide their athletic status; and 19 did not complete any measuring instrument. Furthermore, 12 entries were removed because they were duplicates (based on email addresses) and 20 others were removed because they answered the English version of the questionnaire, resulting in a final sample of 210 participants.

### Measures

#### Sociodemographic questions

At the beginning of the questionnaire, participants reported their age, sex assigned at birth, gender, sexual orientation, athletic status, and principal sport. As a descriptive note, participants’ answers to sex assigned at birth and gender happened to align in this sample. Sports were categorized into five types (team, endurance, technical, weight-category, and esthetic) following Sundgot-Borgen and Larsen (1993). Participants were 18.19 years old on average (*SD* = 2.26). Full demographic data are presented in Table 1.

**Table 1:** Sociodemographic and sport characteristics of the final sample (*n* = 210).

Variables	*n* (%)
Sex assigned at birth	
Women	144 (68.6)
Men	66 (31.4%)
Sexual orientation	
Heterosexual	189 (91.3%)
Sexual minority	18 (8.7%)
Athletic status	
“Relève”	72 (34.3)
“Élite”	103 (49.0)
Quebec Maritimes Junior Hockey League player	14 (6.7)
Type of sport mainly competed in	
Team	84 (40.8)
Endurance	57 (27.7)
Aesthetic	30 (14.6)
Weight categories	16 (7.8)
Technical	19 (9.2)
Played Paralympic discipline	15 (7.1)
Ethnic group	
Canadian	201 (95.7)
First Nations	3 (1.4)
Latin American	4 (1.9)
African American	6 (2.9)
Sub-Saharan African	3 (1.4)
North African	6 (2.9)
Asian	5 (2.4)
Western European	13 (6.2)
Eastern European	6 (2.9)
Attended school	188 (89.5)
Were employed	116 (55.2)

#### Sport psychological safety inventory

The SPSI was used to measure athletes’ perceptions of psychological safety within their current sport environment (Rice et al., 2022). This 11-item self-reported questionnaire validated in English includes a total score and three different subscales: mentally healthy environment (four items), mental health literacy (four items) and low self-stigma (three items). Participants rate the items on a 5-point Likert scale from 0 (*strongly disagree*) to 4 (*strongly agree*). Answers are then added up, with higher scores representing greater psychological safety perceived, a mentally healthy environment, good mental health literacy and low self-stigma, respectively. Item distributions supported the use of ordinal-level confirmatory factor analysis.

The French version of the instrument was developed using a committee-based translation approach, a widely recommended method for cross-cultural adaptation (Sousa and Rojjanasrirat, 2011). First, two bilingual translators independently translated the original (English) inventory to French. Their versions were then reconciled by a committee of translators and sport psychology experts, creating conceptually- and culturally sound preliminary draft. This draft was subsequently translated back into English by two new bilingual translators blind to the original version. Finally, this “back-translation” was compared to the original inventory by the same previous committee to resolve discrepancies and ensure semantic, idiomatic, experiential, and conceptual equivalence. The full French version of the SPSI is available in the online Supplementary material.

#### Coach-athlete relationship questionnaire

To measure the quality of the current coach–athlete relationship, the Coach–Athlete Relationship Questionnaire (CART-Q) was used (Jowett and Ntoumanis, 2004). This measure consists of 11 items and requires participants to indicate how much they agree with each statement on a scale from 1 (*strongly disagree*) to 7 (*strongly agree*). A higher overall score represents a coach-athlete relationship of greater quality. The Cronbach’s alpha and McDonald’s omega values herein demonstrated excellent internal consistency (*α* = 0.93, *ω* = 0.95). The French version was adapted using the same committee-based translation approach as the SPSI.

#### Mental health continuum–short form

To assess participants’ level of mental health in the past month, the Mental Health Continuum–Short Form (MHC-SF) was used. Validated both in English (Keyes et al., 2008) and in French (Doré et al., 2017), this questionnaire requires the participant to indicate how often they have had 14 different (specified) feelings on a scale from 0 (*never*) to 5 (*all the time*). The total score varies between 0 and 70 and represents the overall wellbeing with a higher score indicating a higher level of wellbeing. Cronbach’s alpha and McDonald’s omega values demonstrated excellent internal consistency of the overall scale (*α* = 0.90, *ω* = 0.92) in this sample.

#### Violence toward athletes questionnaire

IV experienced by athletes was assessed using the validated Violence Toward Athletes Questionnaire (VTAQ; Parent et al., 2019), both the athlete (VTAQ-A) and coach (VTAQ-C) version. The athlete version was used to measure athletes’ experiences of IV from their peers since they first began practicing their sport. This self-reported scale comprises nine items assessing peer-perpetrated psychological (4 items), physical (2 items), and sexual (3 items) violence. Responses were recorded on a 4-point Likert scale ranging from 0 (*never*) to 3 (*often; more than 10 times*). Mean scores were computed for each subscale, with higher scores indicating greater exposure to violence. The athlete version demonstrated excellent internal consistency herein (*ω* = 0.94; *α* = 0.90).

Coach-perpetrated violence was assessed using a short form of the VTAQ–C (17 items). This version measures athletes’ experiences of violence by a coach since they first began practicing their sport. Items are rated on the same 4-point Likert scale from (0) *never* to (3) *often; more than 10 times*. The scale includes four dimensions: psychological and neglect (4 items), physical (3 items), sexual (5 items), and instrumental (4 items) violence. The coach version showed excellent internal consistency (*ω* = 0.95; *α* = 0.93).

### Data analysis

A confirmatory factor analysis (CFA), replicating the original three-factor structure of the SPSI, and a measurement invariance across gender test were performed using Mplus version 8.9 (Muthén and Muthén, 1998–2017). In the current sample, responses for sex assigned at birth and gender coincided, so group composition was identical regardless of which variable was selected; gender was used. All other statistical procedures (i.e., internal consistency estimates and correlation analyses) were conducted in R’s 4.4.2 (R Core Team, 2024) psych package (Revelle, 2024). Given the ordinal nature of the SPSI items, the CFA was conducted using the weighted least squares mean, and variance adjusted (WLSMV) estimator, which is designated for categorical data and does not assume multivariate normality. Model fit was evaluated using recommended fit indices and their cutoff (Hu and Bentler, 1999): the root mean square error of approximation (RMSEA; <0.06), the standardized root mean square residual (SRMR; <0.08), the comparative fit index (CFI; >0.90), and the Tucker–Lewis index (TLI; >0.90). When residual covariances were considered, they were retained only if they were both statistically suggested by modification indices and substantively interpretable based on item content. These parameters were included to account for localized dependencies between items with overlapping wording, similar content, or shared methodological features, rather than solely to improve model fit. Internal consistency was assessed using both Cronbach’s alphas and McDonald’s omegas.

Regarding measurement invariance across gender, it was assessed using multigroup CFA. Two models were tested in sequence: a configural model (no equality constraints) and a scalar model (equality constraints on factor loadings and item thresholds). According to Wu and Estabrook (2016), these models are suitable when using WLSMV estimation with ordinal indicators. Invariance across models was evaluated based on changes (Δ) in fit indices; ΔCFI < 0.010 and ΔRMSEA < 0.015 were interpreted as evidence of invariance (Chen, 2007). Latent mean comparisons across gender were also tested for statistical significance using standard errors and *p*-values derived from the multigroup CFA framework.

To evaluate construct validity, Pearson correlation coefficients were computed between SPSI scores and theoretically related variables. The CART-Q and MHC-SF assessed the convergent validity, while the VTAQ assessed the divergent validity.

## Results

### Factor structure

The initial CFA model showed acceptable fit. Based on modification indices and inspection of item content, three residual covariances were retained between Items 1–2, 7–8, and 10–11. These covariances were included to account for localized dependencies between items with potentially overlapping content, rather than solely to improve model fit. The final model demonstrated good fit: RMSEA = 0.076 (90% CI [0.05, 0.10]), SRMR = 0.034, CFI = 0.990, and TLI = 0.986. All factor loadings were statistically significant (*p* < 0.001), ranging from 0.687 to 0.914, supporting the three-factor structure. The standardized factor loadings for the final model are presented in Table 2. As an additional distributional check, floor and ceiling effects were evaluated using the 15% criterion (Terwee et al., 2007). Only 0.4% of participants obtained the minimum (0) and 1.9%, the maximum (44) score, indicating no floor or ceiling effects.

**Table 2:** Factor loadings of the final CFA model of the French SPSI.

Items	Factors		
	Mentally healthy environment	Mental health literacy	Low self-stigma
SPSI item 1	0.886		
SPSI item 2	0.844		
SPSI item 3	0.794		
SPSI item 4		0.842	
SPSI item 5		0.914	
SPSI item 6		0.845	
SPSI item 7		0.763	
SPSI item 8	0.810		
SPSI item 9			0.844
SPSI item 10			0.687
SPSI item 11			0.728

### Internal consistency

The Cronbach’s alpha and McDonald’s omega values demonstrated strong internal consistency for the full French SPSI scale (*α* = 0.87; *ω* = 0.93) and for each of its subscale (mentally healthy environment: *α* = 0.87, *ω* = 0.89; mental health literacy: *α* = 0.87, *ω* = 0.89; low self-stigma: *α* = 0.80, *ω* = 0.81), indicating that the items in a given scale of the French version measure coherent constructs.

### Measurement invariance across gender

The scalar model demonstrated a strong fit (RMSEA = 0.066, CFI = 0.992, TLI = 0.991) and the differences in model fit between the two levels of invariance were negligible (∆CFI < 0.001; ∆TLI = 0.002; ∆RMSEA = −0.008). These results meet the established thresholds for measurement invariance.

### Latent mean comparisons by gender

Comparisons of latent means revealed no statistically significant differences between gender across the three SPSI’s subscales, although a marginal trend (*p* = 0.061) was observed for low self-stigma, with girls reporting slightly lower self-stigma. There was no statistically significant difference in SPSI total scores between boys (*M* = 28.41, *SD* = 7.11) and girls (*M* = 29.55, *SD* = 7.12), *t*(190) = −1.04, *p* = 0.301. Full results from the latent mean comparisons between gender on the three SPSI factors are provided in Table 3.

**Table 3:** Latent mean comparisons across gender on the three SPSI factors.

SPSI factors	Group means difference	SE	*p*
Mentally healthy environment	0.083	0.175	0.637
Mental health literacy	0.005	0.154	0.976
Low self-stigma	0.399	0.213	0.061

### Construct validity

The total score of the French SPSI was significantly positively correlated with the quality of the coach–athlete relationship, as measured by the CART-Q (*r* = 0.39, *p* < 0.001), and with athletes’ overall wellbeing, as measured by the MHC-SF (*r* = 0.41, *p* < 0.001). These associations, albeit moderate, support the French SPSI’s convergent validity. The French SPSI’s total score was also significantly negatively correlated with self-reported experiences of interpersonal violence from peers and authority, as measured by the VTAQ-A (*r* = −0.22, *p* < 0.01) and VTAQ-C (*r* = −0.22, *p* < 0.01). These weak but statistically significant negative correlations provide partial support for divergent validity, suggesting that psychological safety is related to, but not redundant with, athletes’ self-reported experiences of interpersonal violence. The full correlation matrix is presented in Table 4.

**Table 4:** Correlations between key variables.

Variables	1.	2.	3.	4.	5.
SPSI	–				
CART-Q	0.39***	–			
MHC-SF	0.41***	0.19**	–		
VTAQ-A	−0.22**	−0.17*	−0.16*	–	
VTAQ-C	−0.22**	−0.30***	0.18*	0.72***	–

*p < 0.05. **p < 0.01. ***p < 0.001.

## Discussion

The purpose of the present study was to evaluate the psychometric properties of a French version of the SPSI among French-Canadian elite youth athletes. The factor structure and internal consistency met or exceeded established standards, and the expected convergent associations were observed. Evidence for divergent validity was weaker, indicating that this component of the construct warrants further examination but does not alter the robustness of the structural and reliability findings.

The three-factor structure of the Rice et al.’s (2022) original instrument was notably replicated in the present French-Canadian sample. The replication of its three subscales further highlights how, as previously suggested (Walton et al., 2024), “psychological safety” should encompass more than the absence of harm; it includes the proactive support of athlete wellbeing. The replication of the original structurealso suggests that the SPSI and its theoretical model are generalizable to other cultures, at least occidental ones.

Results also demonstrated measurement invariance of the French SPSI across gender, indicating that the instrument functions equivalently for girls and boys in this sample and that the final model applies similarly across these groups. This supports the use of the scale for gender-based comparisons. However, since no participants identified outside the binary categories in this dataset, it limits conclusions regardingthe instrument’s performance for gender-diverse athletes. Gendered dynamics in sport remain relevant for interpreting group differences. Prior research shows that girls more often report exposure to psychological and sexual violence, body-shaming, and discriminatory attitudes (Tuakli-Wosornu et al., 2024; Willson and Kerr, 2023), whereas boys more often report physical violence and may be more affected by norms discouraging emotional disclosure (Tuakli-Wosornu et al., 2024; Wasylkiw and Clairo, 2018). These patterns suggest that while the construct of psychological safety is shared across gender, specific threats to it and the barriers to maintaining it may differ. The invariance of the French SPSI therefore provides a robust tool to investigate such differences empirically, offering new opportunities to examine how gendered dynamics in sport influence athletes’ psychological safety.

The French SPSI’s validity was also assessed through convergent and divergent validity. As hypothesized, there was a positive correlation between psychological safety and psychological wellbeing, as measured by the MHC-SF, indicating that athletes who perceived higher psychological safety also reported greater wellbeing. This is consistent with prior research showing that psychologically safe environments facilitate disclosure, reduce stigma, and foster help-seeking behaviors (Walton et al., 2024). Psychological safety was also positively correlated to the coach–athlete relationship quality, as assessed by the CART-Q, suggesting that a better relationship with coaches is linked to feeling more psychologically safe in sport. The association found with the coach-athlete relationship was also expected, as it aligns with previous studies highlighting the critical role of coaching dynamics in shaping athletes’ perceptions of safety and trust (Davis et al., 2019; Jowett et al., 2023). The moderate correlations between the SPSI-FR and both the MHC-SF and the CART-Q support the former’s convergent validity.

The negative association between psychological safety and interpersonal violence was weaker than expected, suggesting that these constructs are related but not opposite or redundant. Psychological safety should not be interpreted merely as the absence of interpersonal violence. Rather, it reflects athletes’ current perceptions of whether their sport environment allows openness, trust, support-seeking, and expression of concerns without fear of negative consequences. A plausible explanation for the modest association is that the two constructs are anchored to different temporal windows and contextual referents. Psychological safety assesses the current perception of the sport climate, whereas the VTAQ documents violence accumulated across the athlete’s entire sport trajectory, irrespective of the present environment. This temporal mismatch reduces the expected correspondence. It is also likely that normalization dynamics attenuate the link. Research shows that in many competitive settings, athletes learn to minimize, reinterpret, or strategically silence harmful behaviors to avoid social or performance-related consequences (Hughes and Coakley, 1991; Stirling and Kerr, 2009). This reduces the extent to which lifetime reports of violence align with current perceptions of psychological safety, even when the underlying conceptual relation remains intact. Findings from Hurst and Kavussanu (2025) reinforce this interpretation. Their work showed that elite and sub-elite athletes report lower psychological safety but are also less willing to voice interpersonal violence, precisely because these environments cultivate norms that tolerate or normalize harmful behavior and penalize disclosure. Such climates weaken the functional connection between experienced violence and felt safety: past events may have been absorbed into a culture of acceptance that suppresses their impact on present evaluations. This helps explain why, even in a theoretically coherent framework, the association in the current data remains modest.

Taken together, the results indicate that the French SPSI is a psychometrically sound instrument for assessing psychological safety in French-speaking elite youth sport. The confirmed three-factor structure, strong internal consistency, evidence of measurement invariance across gender, and theoretically coherent convergent patterns support its use for research and monitoring purposes, while the weaker association with peer interpersonal violence delineates the limits of its divergent validity rather than undermining the core measure. The SPSI-FR can thus be deployed in French-speaking sport organizations to track the psychosocial climate of teams and clubs, to evaluate the impact of organizational and coaching practices on athletes’ wellbeing, and to identify subgroups of athletes who perceive lower psychological safety and may benefit from targeted support. It is also relevant for talent development programs, sport schools, and high-performance training centers seeking to document and adjust their efforts to build athlete-centered environments.

At a conceptual level, the pattern of findings is consistent with broader safe sport literature. The positive association between psychological safety and wellbeing aligns with work showing that harmful environments and interpersonal violence undermine athletes’ mental health, whereas climates that enable open communication and help-seeking are linked to more adaptive outcomes (Mountjoy et al., 2016; Tuakli-Wosornu et al., 2024; Walton et al., 2024). In line with Walton et al. (2024), the present results are compatible with the view that when athletes feel able to express concerns, seek support without fear of negative consequences, and remain engaged despite adversity, they report both higher wellbeing and greater perceived psychological safety. The SPSI-FR provides a tool to operationalize this construct in French and to examine, with greater precision, how sport environments contribute to or constrain athletes’ psychological health.

### Limitations and directions for future research

Despite its strengths, this study has several limitations. First, given regional variations in the French language, it remains to be verified whether the French Canadian version of the SPSI can be applied without adaptation in other French-speaking regions. As the organization of sport in Canada differs from that in Europe and other francophone contexts, the generalizability of the results should be interpreted with caution. Future research should therefore aim to replicate the validity of the SPSI-FR in other French-speaking cultural contexts and across additional demographic groups. Although the present study supported the validity of the SPSI-FR across girls and boys, its generalizability to populations reflecting greater gender, sexual, and ethnic diversity remains to be established. Second, although the sample included athletes aged 14 to 25 years, the present study did not examine age-related differences or measurement invariance across age subgroups. Given that psychological safety may vary across developmental stages and sport career transitions, future studies should examine whether the SPSI-FR functions equivalently across younger and older athletes. Third, the study relied exclusively on self-report data, which may be influenced by social desirability bias. Although psychological safety inherently involves subjective perceptions, future research should incorporate qualitative and observational designs to complement and contextualize these findings. Fourth, because test–retest reliability was not assessed, the temporal stability of the SPSI-FR remains unknown. Future studies should also assess the SPSI-FR’s sensitivity to change following interventions or policy changes and establish normative scores. Such information would enhance the SPSI-FR’s practical utility and help organizations implement impactful interventions to support elite youth athlete mental health.

## Conclusion

This study contributes to the growing literature on psychological safety in sport by validating a French-Canadian version of the SPSI. The tool demonstrated strong psychometric properties, theoretical alignment with contemporary models of mental health, and relevance to athlete development and protection. As sport systems increasingly prioritize the wellbeing of athletes, tools such as the SPSI are critical to empirically assess, monitor and evaluate the evolution of the state of mental health in the sport world. Nowadays, psychological safety is a cornerstone of mental health. Environments that foster respect, openness, and emotional support (i.e., that respect the very basis of psychologically safe environment) are more likely to produce healthier athletes, but also more sustainable and ethical systems of talent development. The French SPSI represents a significant step toward realizing this vision in a French-speaking environment.

## Data Availability

The datasets generated and/or analyzed during the current study are not publicly available due to participant confidentiality and ethics restrictions, but are available from the corresponding author on reasonable request, subject to approval by the relevant ethics committee. Requests to access the datasets should be directed to veronique.boudreault2@usherbrooke.ca.
