# Supplementary material for: Psychological safety in sport: validation of the French Sport Psychological Safety Inventory (SPSI-FR)
Source: Front Psychol. 2026 Jun 22;17:1855024. doi: 10.3389/fpsyg.2026.1855024 (PMC13333628; doi:10.3389/fpsyg.2026.1855024)
Supplement: Supplementary file 1 [file Supplementary_file_1.DOCX]

Supplementary Material

**SPSI-FR - Sport Psychological Safety Inventory-French Version**

Indiquez votre niveau d’accord avec chacun des items suivants selon l’échelle suivante:

| Fortement en désaccord | En désaccord | Neutre/incertain.e | En accord | Fortement en accord |
| --- | --- | --- | --- | --- |
| 1 | 2 | 3 | 4 | 5 |

| 1. Mon milieu sportif est un endroit sécuritaire pour parler de ses problèmes de santé mentale. | 1 | 2 | 3 | 4 | 5 |
| --- | --- | --- | --- | --- | --- |
| 1. Mon milieu sportif offre un environnement favorable pour parler de ses problèmes de santé mentale. | 1 | 2 | 3 | 4 | 5 |
| 1. Les leaders dans mon sport prennent la santé mentale au sérieux. | 1 | 2 | 3 | 4 | 5 |
| 1. J'ai une bonne connaissance des problèmes de santé mentale en contexte sportif. | 1 | 2 | 3 | 4 | 5 |
| 1. Je connais les signes de problèmes de santé mentale que je dois surveiller en contexte sportif. | 1 | 2 | 3 | 4 | 5 |
| 1. Si je vivais un problème de santé mentale, je serais conscient.e des symptômes. | 1 | 2 | 3 | 4 | 5 |
| 1. Je sais comment maintenir une bonne santé mentale dans un contexte sportif. | 1 | 2 | 3 | 4 | 5 |
| 1. Les personnes de mon milieu sportif me soutiendraient en cas de problèmes de santé mentale. | 1 | 2 | 3 | 4 | 5 |
| 1. Des problèmes de santé mentale pourraient refléter une mauvaise image de moi dans mon milieu sportif. *(R)* | 1 | 2 | 3 | 4 | 5 |
| 1. Je crois que les problèmes de santé mentale en contexte sportif sont signe de faiblesse. *(R)* | 1 | 2 | 3 | 4 | 5 |
| 1. Je penserais avoir échoué si je vivais des problèmes de santé mentale. *(R)* | 1 | 2 | 3 | 4 | 5 |

*Note*. “(R)” means the item needs to be reversed scored. The “mentally healthy environment” subscale consists of items 1, 2, 3, and 8; the “mental health literacy” subscale consists of items 4, 5, 6, and 7; the “low self-stigma” subscale consists of items 9, 10, and 11.
